# Delayed Cord Clamping Increased the Need for Phototherapy Treatment in Infants With AB0 Alloimmunization Born by Cesarean Section: A Retrospective Study

**DOI:** 10.3389/fped.2018.00241

**Published:** 2018-09-19

**Authors:** Stefano Ghirardello, Beatrice L. Crippa, Valeria Cortesi, Elena Di Francesco, Dario Consonni, Lorenzo Colombo, Monica Fumagalli, Arjan B. te Pas, Fabio Mosca

**Affiliations:** ^1^Neonatal Intensive Care Unit, Department of Clinical Sciences and Community Health, Fondazione IRCCS Cà Granda Ospedale Maggiore Policlinico, Università degli Studi di Milano, Milan, Italy; ^2^Epidemiology Unit, Fondazione IRCCS Ca' Granda Ospedale Maggiore Policlinico, Milan, Italy; ^3^Division of Neonatology, Department of Pediatrics, Leiden University Medical Center, Leiden, Netherlands

**Keywords:** jaundice, hyperbilirubinemia, hemolytic disease, AB0 alloimmunization, Rhesus disease

## Abstract

**Objective:** To compare the effect of Delayed Cord Clamping (DCC) to Immediate Cord Clamping (ICC) on phototherapy treatment in a cohort of cesarean-delivered newborns with AB0-alloimmunization.

**Study Design:** In a retrospective cohort study neonates with Gestational Age (GA) ≥ 35 weeks and diagnosed with AB0-alloimmunization before implementation of DCC (ICC group) were compared with neonates born after implementation (DCC group). The primary outcome was the need for phototherapy. Secondary outcomes included hospital stay, readmission rate, need for extra intravenous fluids, maximum bilirubin concentration, and hours of life at bilirubin peak. We used regression models to adjust for weight loss, type of feeding, birth weight, and gestational age.

**Results:** In total 336 neonates were included, of which 192 neonates in the ICC group and 144 in the DCC group. There were no differences in basic characteristics between the two groups except for birth weight (ICC 3193 ± 468 g vs. DCC 3053 ± 446 g, *p* = 0.01) and GA (ICC 38.2 ± 1 weeks of GA, vs. DCC 37.9 ± 1 weeks of GA; *p* = 0.01). When adjusted for confounding factors, after implementation of DCC, significantly more infants with AB0 alloimmunization needed phototherapy (22.4% vs. 36.8%, RR 1.61 CI: 1.15–2.28; *p* = 0.006; Number Needed to Harm 7), needed to stay longer in hospital (20.3% vs. 30.5%, RR 1.53 CI: 1.05–2.23; *p* = 0.03). The maximum bilirubin was higher (11.4 ± 4.0 mg/dl vs. 12.9 ± 3.5 mg/dl, *p* < 0.001) and occurred later [74 (67–92) hours vs. 84 (70–103) hours; *p* = 0.04]. There was no difference in the need for intravenous fluids (1.6% vs. 4.9%; not significant) and readmissions (1.6% vs. 3.5%; not significant).

**Conclusion:** Infants with AB0 alloimmunization needed more often phototherapy and were admitted longer after implementation of DCC policy. Further studies are needed to see whether the benefit of DCC outweighs the increased morbidity, admission days, and related hospital costs.

## Introduction

Delayed Cord Clamping (DCC) in term newborns improves iron stores in infancy, and most international guidelines now recommend at least 30–60 s of DCC in term and preterm newborns not requiring resuscitation (1–3). There are no relevant side effects associated with DCC, except for a 2% increase in term infants requiring phototherapy (4).

Hemolytic Disease of the Newborn (HDN) increases the risk of pathologic jaundice. Rhesus disease is responsible for about 114,000 neonatal deaths and 76,000 cases of kernicterus per year worldwide (5), especially in low-income countries where antenatal anti-D prophylaxis is not guaranteed. Rhesus disease was excluded in most DCC trials, as it was likely that DCC increased the risk for hyperbilirubinemia due to the higher amount of opsonized Red Blood Cells (RBC) transfused from the placenta to the newborn that could undergo hemolysis. In a retrospective study (6) authors observed that 30 s of DCC in a group of neonates with fetal Rh-disease reduced the need for exchange transfusion without increasing the rate of pathologic hyperbilirubinemia when compared to Immediate Cord Clamping (ICC). However, the results were not conclusive due to the differences in Gestational Age (GA), birth weight, mode of delivery and, management of jaundice between the two study periods.

Hemolytic disease of the newborn due to AB0-alloimmunization shares the same biological basis of Rh-disease but is more common and less severe.

From January 2015, our Unit adopted a protocol for the management of umbilical cord clamping; we recommend to clamp the umbilical cord after 1 min from birth in cases of Cesarean-Delivered (CD) term and late preterm newborns not requiring resuscitation. Our study aimed to retrospectively analyze the need for phototherapy in two cohorts of newborns with AB0 alloimmunization, before and after the implementation of DCC in our clinical practice.

## Materials and methods

We conducted a retrospective, single-center cohort study at the baby nursery of the Department of Clinical Sciences and Community Health, Fondazione IRCCS Ca' Granda Ospedale Maggiore Policlinico in Milan, Italy. Ethics committee approval was not required for this non-interventional study.

A protocol for the management of umbilical cord clamping was implemented in January 2015 in our Unit, where cord clamping at 1 min for cesarean (CS) and between 1 and 3 min for vaginally delivered infants (term and late preterm) was recommended. Intravenous oxytocin was given after the cord was clamped. Neonatal caregivers were not standardly present for vaginal deliveries and timing of cord clamping was not formally assessed and recorded. During CS, the timing of cord clamping was observed and recorded by the neonatal caregivers. For this reason, we only included infants born by CS. Before 2015, in CD newborns, the umbilical cord was clamped before 10 s from birth. Milking of the umbilical cord in healthy term and late preterm newborns was not performed in our Unit. Contra-indications to DCC were fetal hydrops, fetal Rh-alloimmunization, maternal HIV or HCV with positive viremia, monochorionic twins and newborns requiring immediate resuscitation.

Per protocol, cord Direct Antiglobulin Test (c-DAT) was performed at each delivery immediately after birth; if positive, we assessed neonatal blood group. Maternal blood group was obtained from obstetric charts. We compared a cohort of neonates who were diagnosed with AB0 alloimmunization and were born by CS before (December 2012 to December 2013; ICC group) and after (October 2015 to October 2017; DCC group) implementation of DCC.

We included in the analysis all neonates admitted to our nursery with GA ≥ 35 weeks since hyperbilirubinemia is uniformly managed in our Unit according to the American Academy of Pediatrics (AAP) guidelines (7). Exclusion criteria for the study were the following: GA < 35 weeks, emergency cesarean section, familial risk factors for pathological jaundice (e.g., spherocytosis, glucose-6-phosphate dehydrogenase deficiency, and Gilbert's syndrome), documented Rh-alloimmunization, neonatal cholestatic jaundice, genetic syndromes, twins, symptomatic polycythemia, hypoglycemia, sepsis, transient tachypnea of newborns, and any case of hospitalization in NICU for reasons unrelated to jaundice.

The primary outcome was hyperbilirubinemia requiring phototherapy during hospitalization. Secondary outcomes were: (1) Hours of phototherapy, (2) Extra-days of hospitalization due to jaundice, defined as each more day of hospitalization following 4 days admission after CS delivery because of phototherapy or bilirubin concentration in the high-risk zone, according to AAP and Bhutani nomograms, (3) Hospital readmission for jaundice, (4) Need for intravenous (IV) Infusion, (5) Immunoglobulin therapy, and (6) Maximum bilirubin concentration (mg/dl). Data were collected from the electronic medical charts (Neocare, I&T Informatica e Tecnologia Srl, Italy), available in our Unit.

We carefully assessed all neonates with positive c-DAT for jaundice, by checking serum bilirubin on capillary gas analysis within the first 12 h of life and subsequently according to clinical needs. The management of hyperbilirubinemia, including the need for phototherapy, IV immunoglobulin, exchange transfusion, and the criteria for phototherapy discontinuation were assessed, according to AAP guidelines (7). Per local protocol, all patients with AB0 alloimmunization were treated with high-intensity phototherapy.

Venous blood samples were not routinely performed in healthy term and late preterm newborns. In the case of hematocrit >70% on capillary samples, we performed a venous cell blood count to check for polycythemia (defined as central venous hematocrit higher than 65% or a hemoglobin value above 22 g/dL).

Before discharge, we estimated the risk of developing severe hyperbilirubinemia according to Buthani nomogram; infants in the “high-risk zone” were monitored for at least 24 h (8). Starting and stopping of phototherapy was retrospectively reviewed by the authors to minimize possible errors in data collection, arising from protocol violations during clinical practice. Clinical and biochemical data collection included: sex, GA, birth-weight, small for gestational age < 3rd percentile according to Bertino's growth charts (9), weight loss (percentage), type of feeding (exclusive breastfeeding, mixed or bottle feeding), East Asian race, and cephalohematoma or significant bruising. We collected the first bilirubin concentration, the bilirubin concentration at Newborn Screening (NS), between 48 and 72 h after birth, the maximum bilirubin concentration, and the hours of life at each determination. Only values of total serum bilirubin or Capillary Bilirubin (CB) were considered.

Categorical variables were expressed as a percentage and quantitative variables as the mean ± Standard Deviation (SD) or median with InterQuartile Range (IQR) in case of non-normal distribution (Shapiro-Wilk test was performed to assess normality of distribution). The two cohorts were compared using the χ^2^-test (or Fisher exact test when expected cell frequency was < 5) for categorical variables and Mann-Whitney *U*-test for quantitative variables. We performed univariate and multiple Poisson regression models with robust standard error to calculate Risk Ratios (RR) with a 95% Confidence Intervals (CI) and Risk Difference (RD) for selected categorical outcomes (10). Linear or quantile regression models were used to calculate mean or median differences between quantitative variables. Multivariable models included gestational age, birth weight, weight loss (0–7%, 7.1–10%, and >10%) and type of feeding (exclusive breastfeeding, mixed, and bottle), all interfering with bilirubin metabolism. For the primary outcome, we calculated the Number Needed to Harm (NNH). Statistical analyses were performed with Stata, version 14 (StataCorp, 2015).

## Result

In total 336 infants were included in the study, of which 192 neonates in the ICC group and 144 in the DCC group. There were no differences in basic characteristics between the two groups except for birth weight (ICC 3193 ± 468 g vs. DCC 3053 ± 446 g, *p* = 0.01) and GA (ICC 38.2 ± 1 weeks of GA, vs. DCC 37.9 ± 1 weeks of GA; *p* = 0.01; Table 1). The number of late preterm and Small for Gestational Age (SGA) newborns were comparable between the two groups.

**Table 1:** Demographic features.

	**AB0 incompatibility**		
	**ICC group (*n* = 192)**	**DCC group (*n* = 144)**	*p*-Value^*^
	**n (%)**	**n (%)**	
**SEX**			
Male	98 (51.0)	68 (47.2)	0.49
Female	94 (49.0)	76 (52.8)	
**GESTATIONAL AGE**			
GA < 37	11 (5.7)	13 (9.0)	0.24
GA ≥37	181 (94.3)	131 (91.0)	
GA (mean ± SD)	38.2 ± 1	37.9 ± 1	0.01
**BIRTH WEIGHT**			
Birth weight (mean ± SD)	3193 ± 468 g	3053 ± 446 g	0.01
SGA < 3rd percentile	5 (2.6)	4 (2.7)	0.92
**WEIGHT LOSS (%)**			
0–7	48 (25.0)	46 (31.9)	0.16
7.1–10	97 (50.5)	69 (47.9)	0.64
>10	47 (24.5)	29 (20.1)	0.35
**TYPE OF FEEDING**			
Exclusive breastfeeding	113 (58.9)	69 (47.9)	0.05
Mixed feeding	63 (32.8)	61 (42.4)	0.07
Bottle feeding	16 (8.3)	14 (9.7)	0.66
**Asian race**	6 (3.1)	5 (3.4)	0.86
Cephalohematoma or significant bruising	1 (0.5)	0 (0.0)	0.38

* *Chi-squared test for categorical variables, Mann–Whitney U-test for quantitative variables. GA, Gestational Age; DCC, Delayed Cord Clamping; ICC, Immediate Cord Clamping; SD, Standard Deviation, SGA, Small for Gestational Age*.

Fifteen neonates in the ICC group (7.8%) and 11 neonates in the DCC group (7.6%) had a capillary hematocrit higher than 70%, but polycythemia was not confirmed by venous blood samples. No patients showed clinical symptoms associated with polycythemia.

After adjusting for gestational age, birth weight, weight loss, and type of feeding, infants receiving phototherapy increased after implementation of DCC (22.4% vs. 36.8% *p* = 0.006; Table 2). The RR for phototherapy in the DCC group was 1.61 (95% CI: 1.15–2.28) with a RD of 14.4% (95% CI: 4.56–24.2) and a NNH of 7. Infants had to stay longer in hospital and the number of extra admission days due to jaundice increased (ICC 20.3% vs. DCC 30.5%; RR 1.53, 95% CI: 1.05–2.23; *p* = 0.03), but there was no difference in infants receiving IV infusion (ICC 1.6% vs. DCC 4.9%; *p* = 0.18), and hospital readmission (ICC 1.6% vs. DCC 3.5%; *p* = 0.21; Table 2). Immunoglobulin infusion was rarely given in both groups (Table 2).

**Table 2:** Outcome.

	**AB0 incompatibility**		**RD (95% CI)**	**RR crude (95% CI)**	RR adjusted^**^ (95% CI)	***p*-Value crude**	***p*-Value adjusted**
	ICC group^*^ (*n* = 192)	**DCC group (*n* = 144)**					
	**n (%)**	**n (%)**					
Phototherapy	43 (22.4)	53 (36.8)	14.4 (4.56, 24.2)	1.64 (1.17, 2.30)	1.61 (1.15, 2.28)	0.004	0.006
Longer hospital stay (Pts)	39 (20.3)	44 (30.5)	10.2 (0.81, 19.7)	1.50 (1.04, 2.18)	1.53 (1.05, 2.23)	0.03	0.03
IV infusion	3 (1.6)	7 (4.9)	3.29 (0.63, 7.22)	3.11 (0.82, 11.8)	2.63 (0.64, 10.8)	0.08	0.18
Hospital readmission	3 (1.6)	5 (3.5)	1.90 (1.56, 5.37)	2.22 (0.54, 9.15)	2.47 (0.60, 10.2)	0.26	0.21
Immunoglobulin	2 (1.0)	1 (0.7)	−0.34 (−2.32, 1.63)	0.67 (0.06, 7.28)	0.54 (0.07, 3.93)	0.73	0.54

* The ICC group was defined as a reference;

** *Risk ratios and 95% confidence intervals from multivariable Poisson regression models with robust standard error adjusted for birth weight, gestational age, weight loss (0–7%, 7.1–10%, and >10%), and type of feeding (exclusive breastfeeding, mixed, and bottle); CI, Confidence Interval; DCC, Delayed Cord Clamping; ICC, Immediate Cord Clamping; IV, Intravenous; Pts, Patients; RD, Risk Difference; RR, Risk Ratio*.

There were no differences in measurements of bilirubin, except for the maximum bilirubin concentration (ICC 11.4 ± 4.0 mg/dl vs. DCC 12.9 ± 3.5 mg/dl; *p* < 0.001; Table 3). We observed a delayed peak of bilirubin in the DCC group (ICC 74 hours, IQR 67–92 vs. DCC 84 hours, IQR 70–103; *p* = 0.04) compared to the ICC group. The ICC group required a longer period of phototherapy compared to DCC (ICC 28 hours, IQR 15–43 vs. DCC 19 hours, IQR 9–51; *p* = 0.07) but the DCC newborns required more extra days of hospital stays (ICC 1 day, IQR 1–3 vs. DCC 2 days, IQR 2–3; *p* = 0.007).

**Table 3:** Measurements.

	**AB0 incompatibility**		**Mean or median difference crude (95% CI)**	Mean or median difference adjusted^**^ (95% CI)	***p*-Value crude**	***p*-Value adjusted**
	ICC^*^ (*n* = 192)	**DCC (*n* = 144)**				
Phototherapy, hours, median (IQR)	28 (15, 43)	19 (9, 51)	−9.0 (−23.3, 5.25)	−10.0 (−21.2, 0.87)	0.21	0.07
1st CB value, mg/dl, mean ± SD	8.8 ± 3.0	8.3 ± 2.8	−0.5 (−1.18, 0.15)	−0.3 (−0.97, 0.37)	0.13	0.38
1st CB value, hours, median (IQR)	47 (30, 67.5)	42 (26, 69)	−5.0 (−17.5, 9.47)	2.0 (−7.69, 11.7)	0.56	0.85
Maximum bilirubin value, mg/dl, mean ± SD	11.4 ± 4.0	12.9 ± 3.5	1.5 (0.73, 2.37)	1.6 (0.78, 2.45)	< 0.0001	< 0.0001
Maximum bilirubin, hours, median (IQR)	74 (67, 92)	84 (70, 103)	10 (−0.64, 20.6)	9 (0.21, 17.8)	0.01	0.04
Bilirubin value at NS, mg/dl, mean ± SD	10.8 ± 3.1	11.1 ± 2.5	0.3 (−0.35, 0.97)	0.4 (−0.26, 1.08)	0.35	0.23
Extra days of hospital stay, days, median (IQR)	1 (1, 3)	2 (2, 3)	1 (−0.15, 1.50)	1 (−0.27, 1.72)	0.11	0.007

* The ICC group was defined as a reference;

** *Mean or median differences and 95% confidence intervals from multivariable linear or quantile regression models adjusted for birth weight, gestational age, weight loss (0–7%, 7.1–10%, and >10%), and type of feeding (exclusive breastfeeding, mixed, and bottle); mean ± standard deviation for normally distributed data; median (IQR) for not normally distributed data; CB, Capillary Bilirubin; CI, Confidence Interval; DCC, Delayed Cord Clamping; dl, deciliter; ICC, Immediate Cord Clamping; IQR, InterQuartile Range; mg, milligrams; NS, Newborn Screening; SD, Standard Deviation*.

## Discussion

Our study compared delayed to immediate umbilical cord clamping in a cohort of cesarean-delivered term and late preterm newborns with AB0 alloimmunization. After adjustment for potential confounding factors, we observed that more infants needed phototherapy and needed to stay longer in the hospital after DCC was implemented. The bilirubin concentration was significantly higher in the DCC group, and the peak occurred later. This is not a new finding, meta-analysis comparing DCC vs. ICC in term infants also demonstrated an increased risk for hyperbilirubinemia and phototherapy (4). It was argued that the benefits at later age outweigh the risk for hyperbilirubinemia. However, in our study, the number of infants needing phototherapy almost doubled and with the extra admission days, this would have a considerable impact on hospital resources and costs. Although phototherapy is safe and a non-invasive therapy, it is possible that we should reconsider the benefits of DCC in infants with AB0-alloimmunization.

HDN is an alloimmune disorder caused by transplacentally transmitted maternal immunoglobulin G antibodies that bind to paternally inherited antigens present on fetal RBC resulting in increased hemolysis, bilirubin concentration, and need for phototherapy (11). HDN due to AB0-incompatibility is more common than Rh-disease but is clinically less severe due to a weaker expression of AB0 antigens at birth. Many researchers excluded Rhesus disease from DCC trials, as there was a common belief that DCC could increase the risk for neonatal jaundice in these patients (4). This effect would have a biological plausibility, as a higher amount of opsonized RBC that passes from the placenta to the newborn could undergo hemolysis.

Garabedian et al. (5) compared DCC with ICC in a cohort study with neonates with Rh-alloimmunization, who were transfused in utero. They reported that the DCC group were less anemic, had higher hemoglobin concentration in the first hour after birth, and required less exchange transfusion postnatally. In contrast to our findings, they observed no differences in maximum bilirubin levels, need for intensive phototherapy and phototherapy duration between the two cohorts. However, it is difficult to interpret the results of Garabedian et al.; the differences in hyperbilirubinemia management, GA and rate of vaginal deliveries likely influenced the outcome. Vaginal delivery is associated with higher hemoglobin values at birth, compared to CD (12), and could explain the higher hemoglobin concentration in the DCC group.

The bilirubin concentration was similar between the cohorts in the first 2 days of life, but maximum bilirubin concentration was significantly higher and occurred later in the DCC group. One possible explanation for this observation could be related to the improved neonatal volemia and urinary output associated with DCC (13, 14); the combined effect of dilution and increased bilirubin urinary excretion in the first days of life may have postponed the peak of bilirubinemia in the DCC group. We speculate that this delayed peak in bilirubin resulted in a lower duration of phototherapy, but also prolonged the admission in the hospital. Although more infants needed phototherapy, the duration was shorter as the threshold for phototherapy would be higher after the first days after birth when the bilirubin peaked later. However, this would also lead to a longer stay in the hospital.

Our results suggest that DCC in infants with hemolytic jaundice could increase morbidity. AB0 alloimmunization could be considered a *proxy* for Rh alloimmunization since they share a common pathophysiological basis. Rhesus disease affects about 370,000 newborns worldwide with approximately 114,000 cases of neonatal deaths and 76,000 cases of kernicterus per year, most of them occurring in low-income countries (6). Given the morbidity and mortality of Rhesus disease, randomized trials are needed to assess the safety of delayed cord clamping in this population.

Our study has certain limitations and caution is needed when interpreting the results. Hemoglobin level after birth was not routinely evaluated and the effect of placental transfusion on hemoglobin and hematocrit could not be retrospectively evaluated. We are thus unaware of the incidence of asymptomatic polycythemia in our cohort.

However, according to our knowledge, the meta-analysis by Mc Donald et al. (five trials) reported no difference in the incidence of polycythemia between the early and late cord clamping groups (RR 0.39 95% CI 0.12 to 1.27; 1025 infants) (4) and authors did not specifically recommend to check DCC newborns for polycythemia.

Differently, Hutton et al. reported an higher incidence of asymptomatic polycythemia associated with delayed cord clamping in term (15) newborns, without an increased risk for phototherapy or partial exchange transfusion.

The lower birth weight in the DCC group does not confirm an increase in placental volume. Although there was no difference in the number of late preterm between groups, infants in the DCC group were slightly younger, and probably influenced the difference in birth weight.

Animal models and clinical studies showed a reduced placental transfusion after cesarean delivery (16–18); however, Andersson et al. (19) have recently demonstrated that 30-s DCC in elective CD newborns resulted in similar iron status at 4 months of age if compared to 3-min DCC in vaginal deliveries. As we included only infants born by CS, it is possible that we underestimated the effect of DCC on phototherapy. Also, immaturity could have played a role; however, late preterm neonates were equally distributed in the two groups.

The DAT has a poor positive predictive value for identifying newborns at risk of clinically significant hyperbilirubinemia (20), and a positive DAT does not mean that increased hemolysis will occur. Suggested criteria for HDN are blood group incompatibility plus the demonstration of hemolysis (elevate reticulocyte count and lactic dehydrogenase enzyme concentration, progressive anemia, transient microspherocytosis) and a positive DAT (20). However, elevated reticulocyte count and microspherocytosis are common hematological features in the first days of life, and their interpretation is not univocal. Serial venous blood samples for full blood examination may be too invasive to document progressive anemia in a nursery setting. Therefore, it is a prevailing attitude in clinical practice to consider an infant with a positive DAT to be at higher risk for significant jaundice. AAP charts for the management of neonatal hyperbilirubinemia (7) reduced the threshold for treatment in positive DAT newborns and Bhutani Nomogram (8) for the designation of the risk of hyperbilirubinemia at discharge considered AB0 incompatibility as a risk factor for significant jaundice. If future studies confirm the association between DCC and a delayed peak of bilirubin in AB0 immunization, the nomograms for the designation of the risk of significant hyperbilirubinemia may need to be revised.

## Conclusion

To our knowledge, this retrospective study is the first that analyzed the effect of DCC in a cohort of infants at increased risk for pathological jaundice due to AB0 alloimmunization. In infants with AB0 alloimmunization, we observed an association between DCC, need for phototherapy, and prolonged admission. Since we did not include VD, where the effect of placental transfusion is larger, it is possible we underestimated the effect of DCC. Adequately designed studies in a randomized setting are needed to confirm or refute our findings, but centers should balance the benefit of DCC against the increased risk, resources and costs, especially in infants with alloimmunization.

## Author contributions

SG designed the study, supervised data collection, contributed to interpretation of results, drafted the initial manuscript and reviewed the final manuscript. BC collected data, contributed to interpretation of results, drafted the initial manuscript and reviewed the final manuscript. VC and ED collected data, contributed to interpretation of results and reviewed the final manuscript. DC carried out the statistical analysis, contributed to interpretation of results and reviewed the final manuscript. LC and MF supervised data collection and reviewed the final manuscript. AtP contributed to interpretation of results, reviewed and revised manuscript. FM supervised data collection, contributed to interpretation of results and reviewed the final manuscript. All authors contributed to manuscript revision, read and approved the submitted version.

### Conflict of interest statement

The authors declare that the research was conducted in the absence of any commercial or financial relationships that could be construed as a potential conflict of interest.
